# GTSE1: A potential prognostic and diagnostic biomarker in various tumors including lung adenocarcinoma

**DOI:** 10.1111/crj.13757

**Published:** 2024-05-07

**Authors:** Guanqiang Yan, Guosheng Li, Xiang Gao, Jun Liu, Yue Li, Jingxiao Li, Huafu Zhou

**Affiliations:** ^1^ Department of Cardio‐Thoracic Surgery First Affiliated Hospital of Guangxi Medical University Nanning Guangxi People's Republic of China

**Keywords:** G2 and S phase‐expressed‐1, immune infiltration, lung adenocarcinoma, pan‐cancer, prognostic biomarkers

## Abstract

**Objective:**

This research was aimed to comprehensively investigate the expression levels, diagnostic and prognostic implications, and the relationship with immune infiltration of G2 and S phase‐expressed‐1 (*GTSE1*) across 33 tumor types, including lung adenocarcinoma (LUAD), through gene expression profiling.

**Methods:**

*GTSE1* mRNA expression data together with clinical information were acquired from Xena database of The Cancer Genome Atlas (TCGA), ArrayExpress, and Gene Expression Omnibus (GEO) database for this study. The Wilcoxon rank‐sum test was used to detect differences in *GTSE1* expression between groups. The ability of *GTSE1* to accurately predict cancer status was evaluated by calculating the area under the curve (AUC) value for the receiver operating characteristic curve. Additionally, we investigated the predictive value of *GTSE1* in individuals diagnosed with neoplasms using univariate Cox regression analysis as well as Kaplan–Meier curves. Furthermore, the correlation between *GTSE1* expression and levels of immune infiltration was assessed by utilizing the Tumor Immune Estimate Resource (TIMER) database to calculate the Spearman rank correlation coefficient. Finally, the pan‐cancer analysis findings were validated by examining the association between *GTSE1* expression and prognosis among patients with LUAD.

**Results:**

*GTSE1* exhibited significantly increased expression levels in a wide range of tumor tissues in contrast with normal tissues (*p* < 0.05). The expression of *GTSE1* in various tumors was associated with clinical features, overall survival, and disease‐specific survival (*p* < 0.05). In immune infiltration analyses, a strong correlation of the level of immune infiltration with the expression of *GTSE1* was observed. Furthermore, *GTSE1* demonstrated good discriminative and diagnostic value for most tumors. Additional experiments confirmed the relationship between elevated *GTSE1* expression and unfavorable prognosis in individuals diagnosed with LUAD. These findings indicated the crucial role of *GTSE1* expression level in influencing the development and immune infiltration of different types of tumors.

**Conclusions:**

*GTSE1* might be a potential biomarker for the prognosis of pan‐cancer. Meanwhile, it represented a promising target for immunotherapy.

## Graphical abstract

The study explored *GTSE1* expression levels, diagnostic and prognostic significance, and its association with immune infiltration across 33 cancer types. Using data from TCGA, ArrayExpress, and GEO databases, *GTSE1* was found to be significantly overexpressed in various tumors, correlating with clinical features and survival rates, suggesting its potential as a biomarker for pan‐cancer prognosis and a target for immunotherapy.

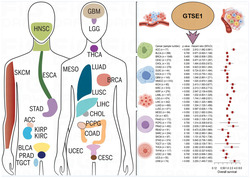

**Abbreviations**
ACC: adrenocortical carcinoma
AJCC: American Joint Committee on Cancer
AUC: area under the curve
BLCA: bladder urothelial carcinoma
BRCA: breast invasive carcinoma
CESC: cervical squamous cell carcinoma and endocervical adenocarcinoma
CHOL: cholangiocarcinoma
CI: confidence interval
COAD: colon adenocarcinoma
DFI: disease‐free interval
DLBC: lymphoid neoplasm diffuse large b‐cell lymphoma
DSS: disease‐specific survival
ESCA: esophageal carcinoma
GBM: glioblastoma multiforme
GSEA: gene set enrichment analysis
HNSCC: head and neck squamous cell carcinoma
HR: hazard ratio
IHC: immunohistochemistry
KEGG: Kyoto Encyclopedia of Genes and Genomes
KICH: kidney chromophobe
KICP: kidney renal papillary cell carcinoma
KIRC: kidney renal clear cell carcinoma
LAML: acute myeloid leukemia
LGG: brain lower grade glioma
LIHC: liver hepatocellular carcinoma
LUAD: lung adenocarcinoma
LUSC: lung squamous cell carcinoma
MESO: mesothelioma
mRNA: messenger RNA
MSI: microsatellite instability
OS: overall survival
OV: ovarian serous cystadenocarcinoma
PAAD: pancreatic adenocarcinoma
PCPG: pheochromocytoma and paraganglioma
PFI: progression‐free interval
PRAD: prostate adenocarcinoma
READ: rectum adenocarcinoma
ROC: receiver operating characteristic
SARC: sarcoma
SKCM: skin cutaneous melanoma
SMD: standardized mean difference
sROC: summary receiver operating characteristic
STAD: stomach adenocarcinoma
TCGA: The Cancer Genome Atlas
TGCT: testicular germ cell tumors
THCA: thyroid carcinoma
THYM: thymoma
TMB: tumor mutation burden
UCEC: uterine corpus endometrial carcinoma
UCS: uterine carcinosarcoma
UVM: uveal melanoma

## 1 INTRODUCTION

The global incidence of cancer is approximately 20 million cases annually, resulting in nearly 10 million deaths. Breast cancer (BRCA) has surpassed lung cancer as the most prevalent type since 2020, accounting for around 2.3 million new cases (11.7%). Lung cancer (11.4%) and colorectal cancer (10.0%) closely follow in terms of mortality. However, lung cancer remains the leading cause of mortality, with 8 million deaths (18%), significantly higher than colorectal cancer (9.4%), liver cancer (8.3%), stomach cancer (7.7%), and female BRCA (6.9%).^1^ Currently, a significant number of patients with lung cancer were diagnosed in advanced stages, which poses considerable treatment challenges. Therefore, early diagnosis is of immense significance, especially for screening individuals at higher risk, such as smokers and those exposed to smoke, oil fields, or toxic occupational environments.^2^ Histological subtypes of lung cancer include adenocarcinoma, squamous cell carcinoma, large cell carcinoma (also known as non‐small cell lung cancer), and small cell lung cancer. The extensive analysis of molecular features in lung adenocarcinoma (LUAD) has broadened our understanding of the cellular foundations and intricate molecular pathways associated with each subtype. Several genetic alterations have been identified as potential therapeutic targets and are currently under continuous development.^3^ In recent years, there have been notable advancements in the identification, description, and management of LUAD.^4^ Comprehensive genomic, epigenomic, and cellular analyses of LUAD and the tumor microenvironment have contributed to a better understanding of its development.^5^ Researchers had continuously discovered pan‐cancer related genes and developed potential targets for tumor diagnosis and treatment through pan‐cancer analysis network.^6^, ^7^, ^8^ However, the lack of corresponding targeted drug therapies has limited their widespread application. Therefore, further exploration of more effective markers for the diagnosis and treatment of LUAD is of utmost importance to facilitate early detection and reduce patient mortality.

G2 and S phase‐expressed‐1 (*GTSE1*) is a gene induced by the p53 protein, located on chromosome 22q13.2‐q13.3, which exhibits specific expression during the S and G2 stages of the cell cycle.^9^ *GTSE1* preferentially binds to the most stable microtubules (MT) of the mitotic spindle and promotes their turnover.^10^ In non‐diploid carcinoma cell lines and tumors, overexpression of *GTSE1* regulates MT stability during mitosis by inhibiting the activity of MCAK, an MT depolymerase enzyme.^11^ *GTSE1* has been implicated in various malignant tumors, including osteosarcoma, BRCA, and colon cancer, regarding their occurrence, development, and prognosis.^12^, ^13^, ^14^ Lai et al.^15^ found that upregulation of *GTSE1* can stimulate the growth of prostate cancer cells via the SP1/FOXM1 signaling pathway, while Zhang et al.^16^ suggested that *GTSE1* induces chromosomal instability in esophageal squamous cell carcinoma cells and inhibits cell apoptosis through the ROS/JNK signaling pathway. Furthermore, enhancing *GTSE1* expression facilitates the growth and spread of LUAD cells through the activation of alternative signaling pathways, such as the AKT/mTOR and ERK/MAPK pathways.^17^, ^18^ *GTSE1* also confers radio‐resistance to LUAD by promoting clonogenic generation and inhibiting cell apoptosis. Knocking down *GTSE1* expression significantly reduces the proliferation and metastatic potential of LUAD cells while enhancing their sensitivity to radiotherapy.^19^ Although more and more evidences support the significant involvement of *GTSE1* in the development of different carcinoma types, most studies had focused on individual cancers, lacking a systematic pan‐cancer analysis of *GTSE1*. Therefore, it was crucial to explore the association of *GTSE1* expression levels, prognosis of patients, and tumor immune infiltration levels across various tumor types, including LUAD.

The aim of this study was to comprehensively analyze the degree of *GTSE1* expression, along with its diagnostic and prognostic value in 33 types of malignant tumors, as well as its correlation with immune invasion using gene expression profiles. Additionally, we conducted further investigations to validate the specific expression pattern of *GTSE1* in LUAD.

## 2 MATERIAL AND METHODS

### 2.1 Pan‐cancer expression data collection

The Cancer Genome Atlas (TCGA, https://www.portal.gdc.cancer.gov/) database encompasses a wide range of information on various types of human cancers, including clinical data, genomic variations, mRNA expression levels, miRNA expression patterns, and methylation profiles. To obtain the mRNA expression data for *GTSE1* in the TCGA dataset, which includes 33 cancer types, we retrieved it from the Xena database. The mRNA expression values of GTSE1 were processed using the log2 (x + 1) conversion method, aiming to mitigate data skewness and promote a closer adherence to the normal distribution, thereby facilitating subsequent analyses.^20^, ^21^

### 2.2 Collection of clinical parameters and prognostic data of pan‐cancer

Critical clinical characteristics for cancer patients, including age, gender, and the American Joint Committee on Cancer (AJCC) stage, were obtained from the TCGA dataset for the 33 cancer types. The Xena database was utilized to retrieve this information. Additionally, prognosis data, including overall survival and disease‐specific survival time, were collected from the same database.

### 2.3 Analysis of immune infiltration levels across pan‐cancer

The Tumor Immune Estimation Resource (TIMER, https://cistrome.shinyapps.io/timer/) database^22^, ^23^ utilizes high‐throughput sequencing technology (RNA‐Seq expression profiling) to evaluate the infiltration of immune cells within tumor tissues. It primarily provides insights into B cells, CD4+ T cells, CD8+ T cells, neutrophils, macrophages, and dendritic cells. Besides, Estimation of STromal and Immune cells in MAlignant Tumours using Expression data (ESTIMATE)^24^ is an alternative approach for assessing immune infiltration, enabling the determination of the immune interstitial score, ESTIMATE score, and tumor purity in a given sample. In this study, we employed both TIMER and ESTIMATE techniques to assess the levels of immune infiltration in tumor samples, aiming to explore the potential association of *GTSE1* expression with immune infiltration levels.

### 2.4 LUAD expression and prognostic data acquisition

To partly validate the main findings in pan‐cancer, we conducted additional analysis focusing on *GTSE1* in LUAD. Public LUAD mRNA expression data were obtained from various databases, including ArrayExpress (https://www.ebi.ac.uk/biostudies/arrayexpress) and Gene Expression Omnibus (GEO, https://www.ncbi.nlm.nih.gov/geo/). The inclusion criteria were as follows: (1) LUAD tissue samples collected from patients with LUAD, (2) paired samples of tissues adjacent to cancer included in the same dataset, and (3) complete mRNA expression data. The exclusion criteria were as follows: (1) non‐human tissue samples and (2) incomplete *GTSE1* expression data. Ultimately, nine LUAD datasets, consisting of 485 LUAD samples and 485 samples adjacent to LUAD tissues, were collected for the analysis. The acquisition of prognostic data allowed us to evaluate the prognosis and survival outcomes of LUAD patients, facilitating the development of personalized treatment strategies. For this purpose, four prognostic datasets (GSE11963, GSE13213, GSE14814, and GSE19188), including 377 patients with LUAD, were collected to explore the correlation between *GTSE1* expression and the prognosis of LUAD patients.

### 2.5 Statistical analysis

The Wilcoxon rank‐sum test was employed to identify any variations in the expression of *GTSE1* between groups.^25^ Spearman's rank correlation coefficients were used to assess the association between the expression of *GTSE1* and immune infiltration levels. The predictive accuracy of *GTSE1* in determining cancer status was evaluated by calculating the area under the curves (AUC) using receiver operating characteristic (ROC) analysis. The prognostic value of *GTSE1* on patients with malignancies was evaluated utilizing univariate Cox regression analysis and Kaplan–Meier curves. Statistical analyses were conducted by R software (v4.1.0), except for the generation of the summary ROC curve, which was conducted in Stata (version 15.0). A *p* value below 0.05 was considered statistically significant.

## 3 RESULTS

### 3.1 The differential expression of *GTSE1* between cancer tissues and control tissues

**FIGURE 1:**
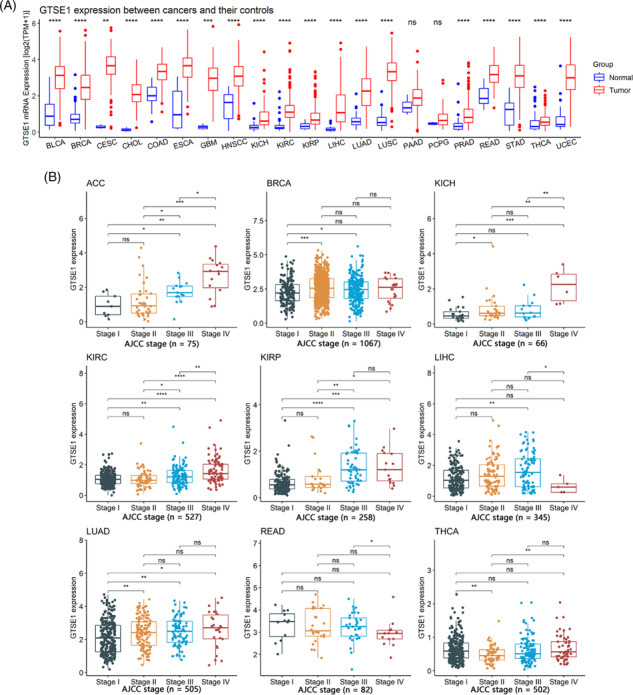
The expression of *GTSE1* and its correlation with tumor stages in cancers. (A) Differential expression of *GTSE1* mRNA between cancers and controls; *p* value was based on the Wilcoxon rank‐sum test. (B) Relation of *GTSE1* expression with tumor stages. ^ns/NS^ *p* > 0.05; *p < 0.05; ^**^ *p* < 0.01; ^***^ *p* < 0.001; ^****^ *p* < 0.0001. AJCC, American Joint Committee on Cancer.

This study, comprising 9358 tumor samples and 722 control samples, undertook a comprehensive investigation into the differential expression of GTSE1 between cancerous and control tissues across diverse human tumor types. The findings revealed distinct *GTSE1* expression levels among different cancer tissues in contrast with control tissues. Notable disparities were observed in 19 tumor types, such as bladder urothelial carcinoma, where the mRNA expression level of *GTSE1* was higher among cancer tissues compared to the control tissues with statistical significance (*p* < 0.05; Figure 1A). However, no significant differences in *GTSE1* expression were detected in cases of pancreatic adenocarcinoma (PAAD) and pheochromocytoma and paraganglioma (PCPG) when comparing cancer tissues to control tissues (*p* > 0.05; Figure 1A). Therefore, *GTSE1* exhibits high expression in most tumors.

### 3.2 The relationship between *GTSE1* and clinical parameters in tumor patients

**FIGURE 2:**
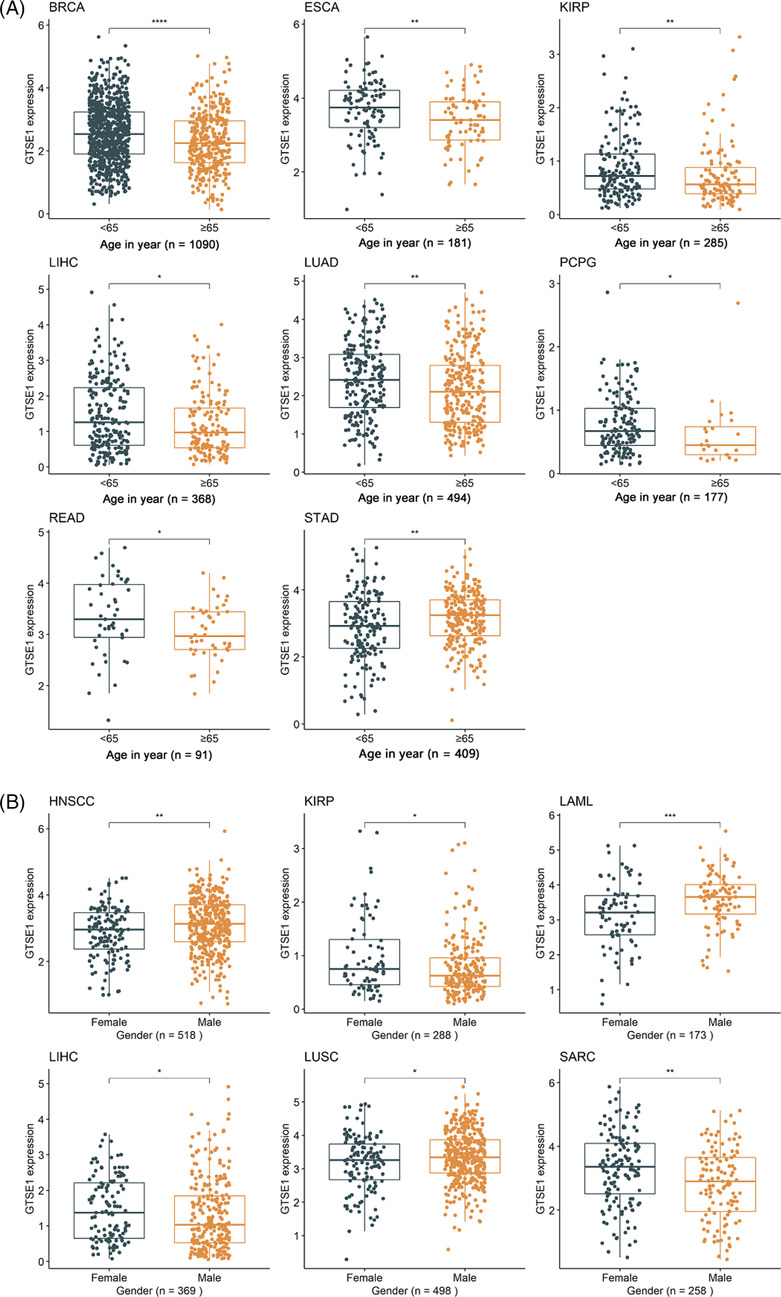
Relation of *GTSE1* expression with ages (A) and gender (B).

The differential expression of *GTSE1* in patients with different clinical parameters was determined using Wilcoxon rank‐sum tests. The results showed that different AJCC stages were associated with varying levels of *GTSE1* expression in nine different tumors (*p* < 0.05; Figure 1B), including adrenocortical carcinoma (ACC), breast invasive carcinoma (BRCA), kidney chromophobe (KICH), kidney renal clear cell carcinoma (KIRC), kidney renal papillary cell carcinoma (KIRP), liver hepatocellular carcinoma (LIHC), LUAD, rectum adenocarcinoma (READ), and thyroid carcinoma (THCA). Higher AJCC stages were consistently associated with higher levels of *GTSE1* expression. Moreover, younger patients (<65 years old) with BRCA, esophageal carcinoma (ESCA), KIRP, LIHC, LUAD, PCPG, and READ had higher levels of *GTSE1* expression in cancer tissues (*p* < 0.05; Figure 2A). Conversely, STAD (stomach adenocarcinoma) showed lower levels of *GTSE1* in younger patients (<65 years old) (*p* < 0.05; Figure 2A). In HNSCC (head and neck squamous cell carcinoma), LAML (acute myeloid leukemia), and LUSC (lung squamous cell carcinoma), male patients exhibited higher levels of *GTSE1* expression compared to female patients. However, male patients with KIRP, LIHC, and SARC (sarcoma) had reduced levels of GTSE1 expression (*p* < 0.05; Figure 2B). Baseline information from the TCGA dataset (Supporting Information S1) alongside the results of multivariate Cox regression analysis (Supporting Information S2) aids in investigating the relationship between GTSE1 and overall cancer prognosis, elucidating its prognostic significance across multiple cancer types.

### 3.3 The association between *GTSE1* expression and the prognosis of cancer patients

**FIGURE 3:**
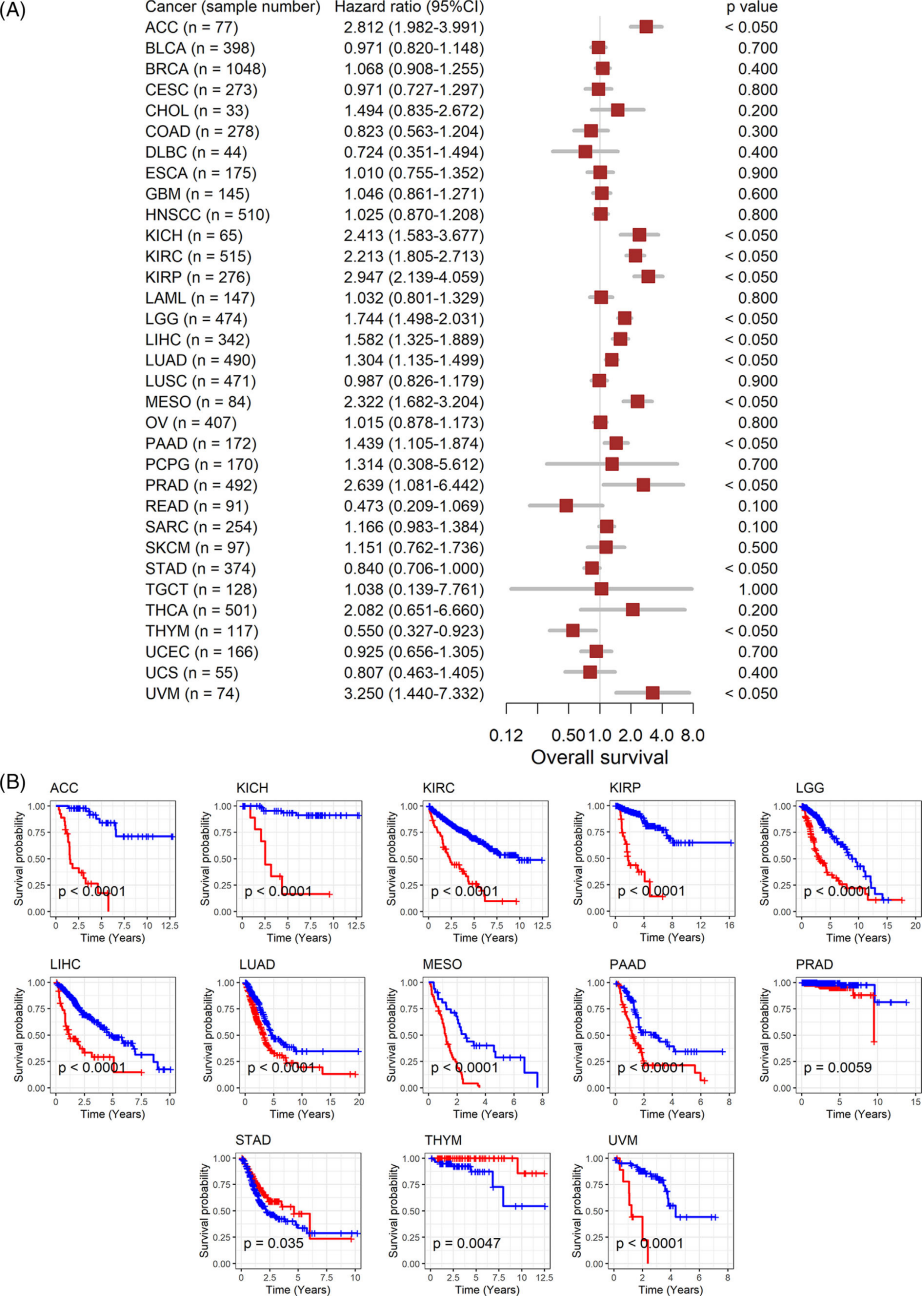
Relation of *GTSE1* expression with overall survival of cancer patients. (A) Univariate Cox regression analysis. (B) Kaplan–Meier curves. *Note*: GTSE1 expression values used for the calculation of Figure 3A are considered as continuous variables.

**FIGURE 4:**
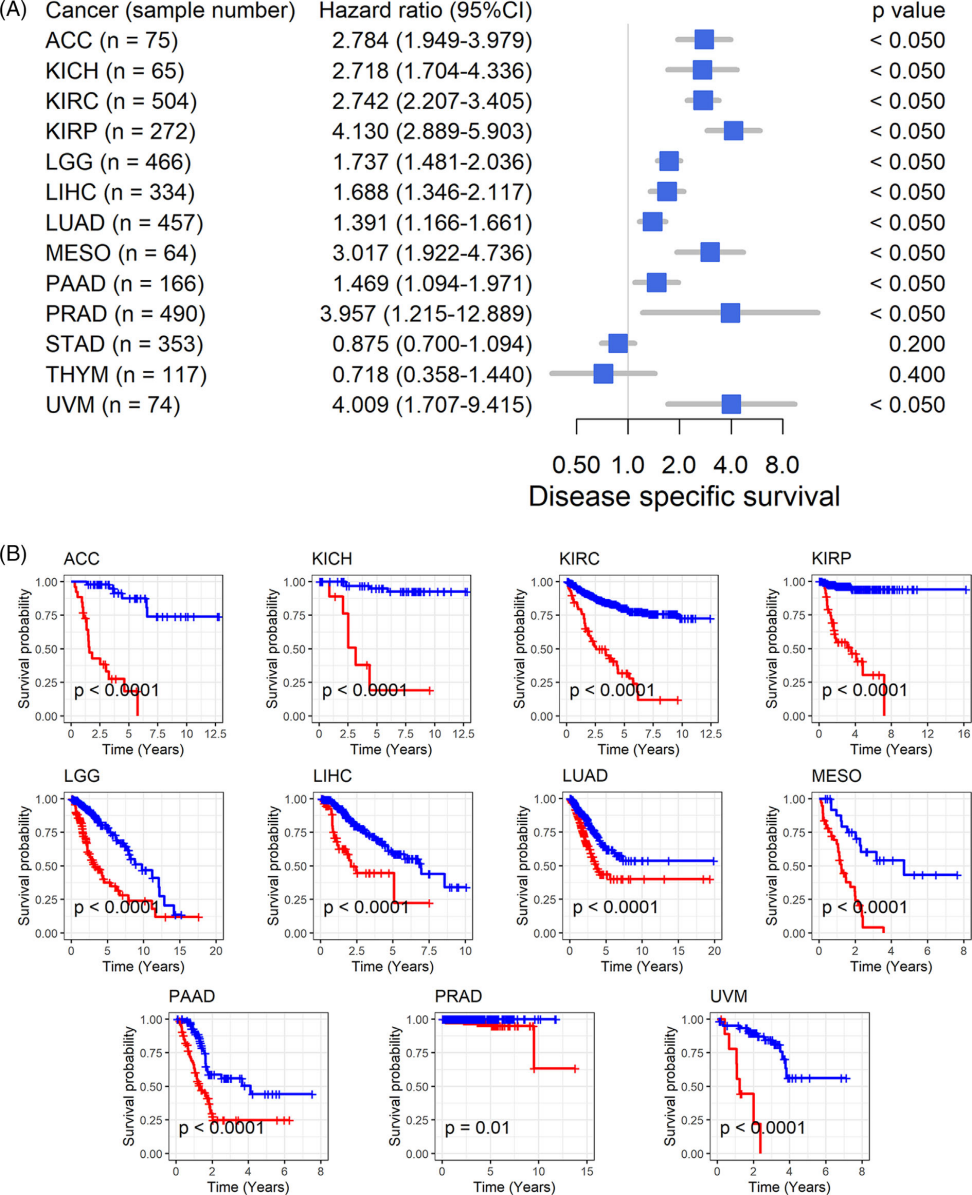
Relation of *GTSE1* expression with and disease‐specific survival of cancer patients. (A) Univariate Cox regression analysis. (B) Kaplan–Meier curves. *Note*: GTSE1 expression values used for the calculation of Figure 4A are considered as continuous variables.

The association between *GTSE1* expression and the prognosis of cancer patients suggests further analysis of its correlation with patient prognosis. High expression of *GTSE1* is consistently linked to poor overall survival in 11 types of tumors, including ACC, indicating it as a risk factor (HR > 1, *p* < 0.05; Figure 3A) associated with shorter survival time for patients (*p* < 0.05; Figure 3B). Conversely, in STAD and THYM patients, high *GTSE1* expression acts as a protective factor for overall survival (HR < 1, *p* < 0.05; Figure 3A), leading to extended survival time (*p* < 0.05; Figure 3B). Disease‐specific survival data further confirm these results in the overall survival analysis for the remaining 11 cancer types (HR > 1, *p* < 0.05; Figure 4A,B). Based on these findings, it could be inferred that the elevated *GTSE1* expression is a significant prognostic hazard factor.

### 3.4 Potential identification effect of tumor patients using *GTSE1*

**FIGURE 5:**
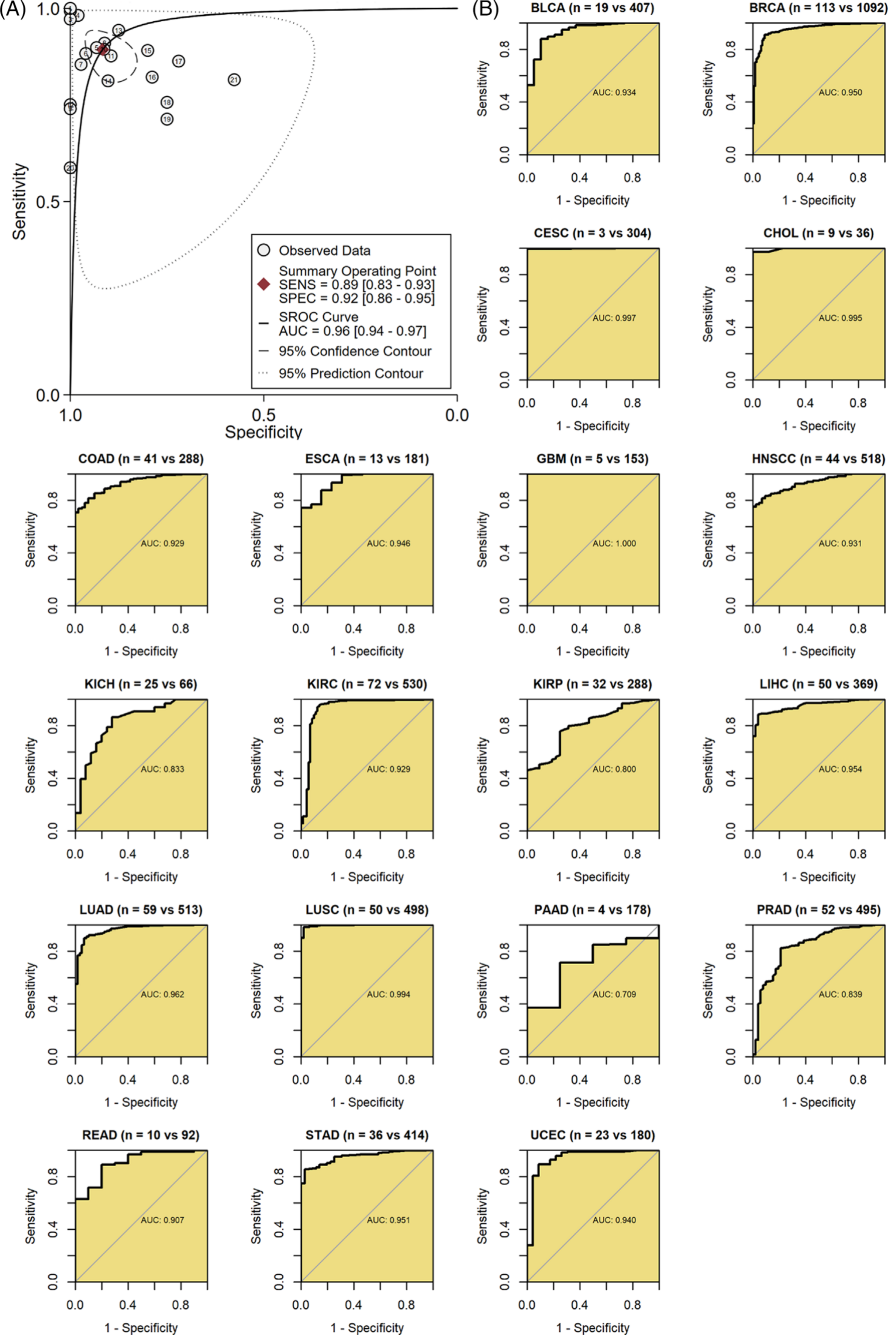
The ability of *GTSE1* to differentiate the tumor tissue from control tissue. (A) The summary receiver operating characteristic curve shows that *GTSE1* distinguishes cancers well from control tissues in 19 cancer types. (B) Summary receiver operating characteristic curves shows that *GTSE1* can accurately distinguish cancer tissues from control tissues in 19 cancers.

It is crucial for a clinical biomarker to distinguish tumor patients. In this study, results demonstrated that *GTSE1* expression effectively distinguished cancer tissues from normal tissues in the 21 tumor types included in this study (sensitivity = 0.89, specificity = 0.92, area under the curve = 0.96; Figure 5A). In the ROC analysis of individual tumor types, AUC > 0.7 was observed for 19 types of cancer, indicating that *GTSE1* mRNA expression levels are highly potential to differentiate cancer tissues from control tissues in these tumors (Figure 5B).

### 3.5 Relationship of *GTSE1* expression and immune infiltration in tumors

**FIGURE 6:**
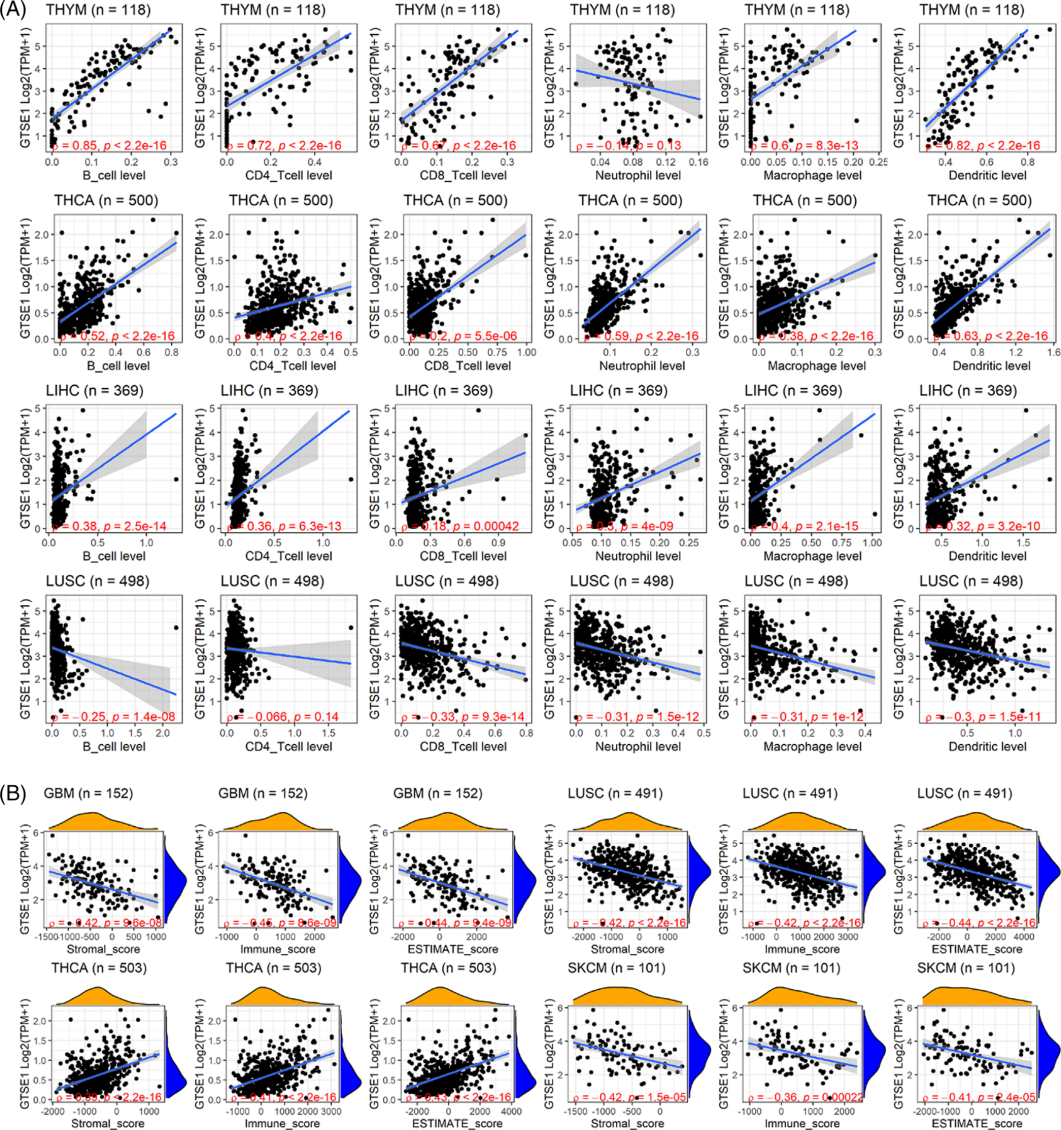
Correlation of *GTSE1* expression and immune cell infiltration levels in several cancer types. (A) TIMER algorithm; (B) ESTIMATE algorithm.

Immune infiltration plays a crucial role in tumor initiation and progression, holding significant clinical significance in various tumors. Thus, this study investigated the relationship of *GTSE1* and tumor immune infiltration. Based on the results from the TIMER algorithm, the expression level of *GTSE1* was found to be positively correlated with the infiltration levels of six immune cells in THYM, THCA, and LIHC (*p* < 0.05; Figure 6A). In LUSC, a negative correlation was observed between *GTSE1* expression level and the infiltration levels of six immune cells (*p* < 0.05; Figure 6A). Another algorithm, ESTIMATE, revealed a negative correlation between *GTSE1* expression level and three immune scores in GBM, LUSC, and SKCM, while it showed a positive correlation with the immune score in THCA (*p* < 0.05; Figure 6B).

### 3.6 Expression and clinical implications of *GTSE1* in LUAD

**FIGURE 7:**
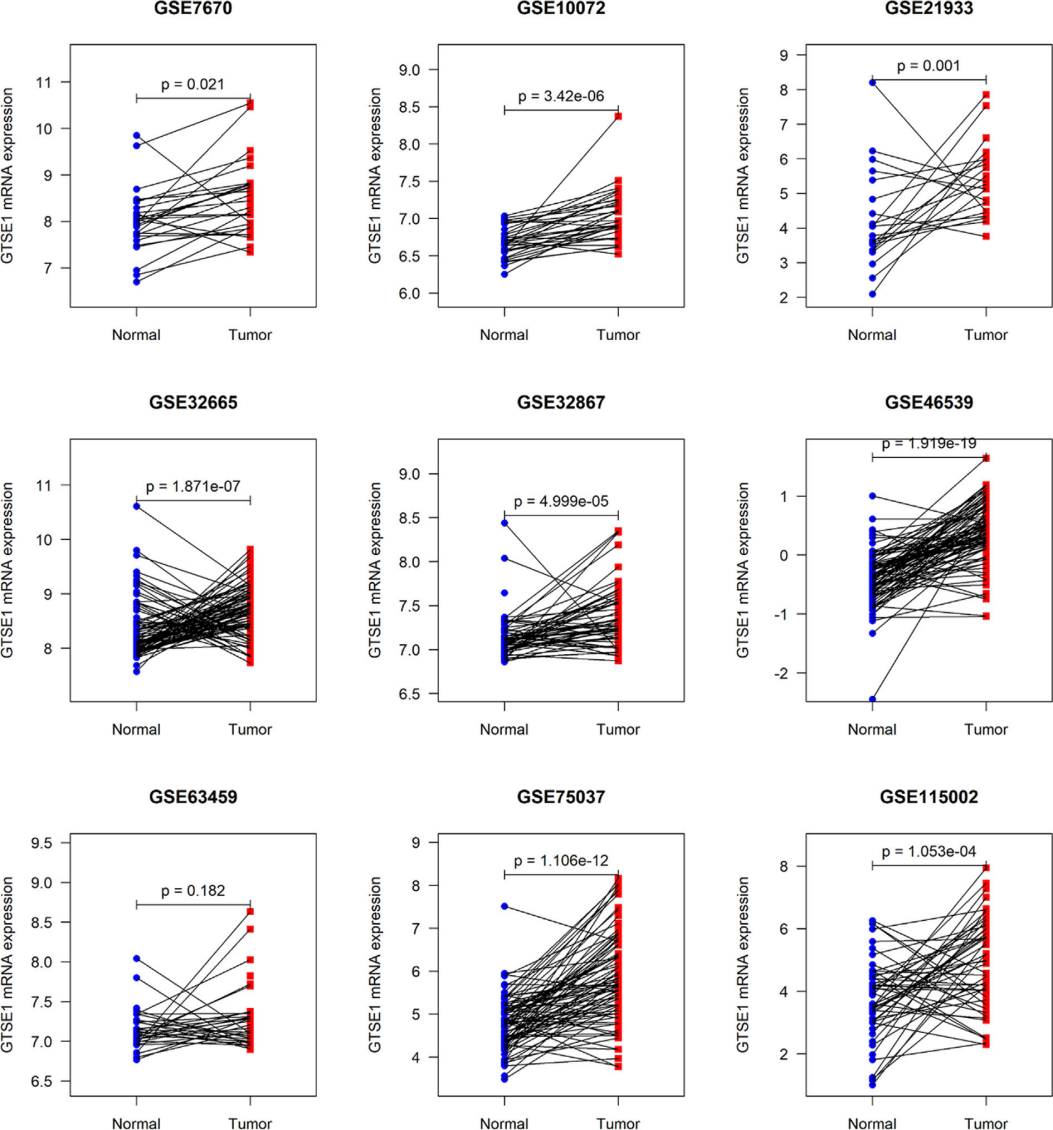
The difference of *GTSE1* expression between LUAD and control groups using multi‐center data. *P* value in the plot was based on the paired *t* test.

**FIGURE 8:**
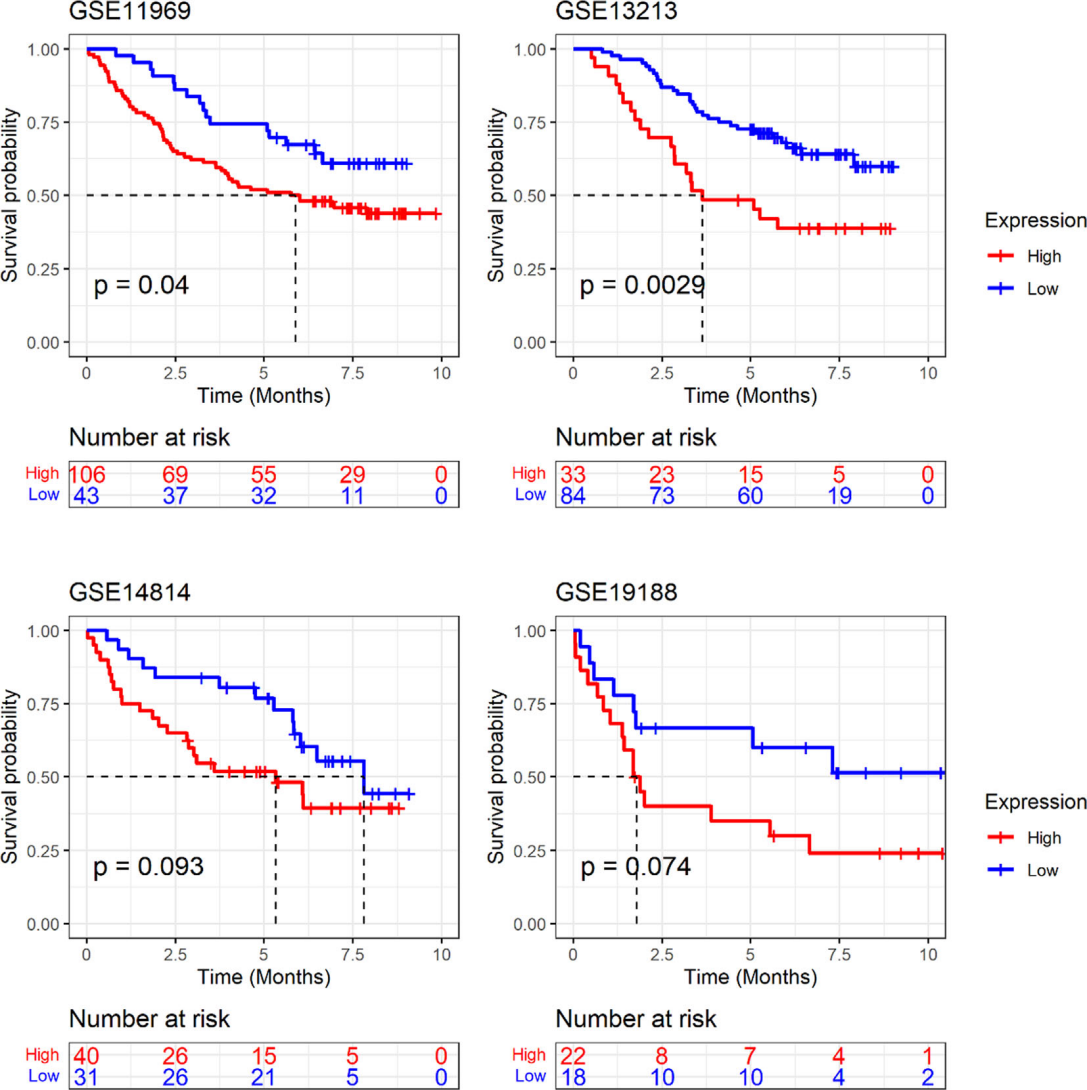
The effect of *GTSE1* expression on the prognosis of patients with lung adenocarcinoma.

Currently, lung cancer has been reported to have the highest mortality rate among tumors. Using LUAD as an example, this study further verified the expression and clinical relevance of *GTSE1*. Analysis of multi‐center data showed a higher expression level of *GTSE1* mRNA in LUAD tissue compared to that in normal lung tissue (*p* < 0.05; Figure 7). Similar to most cancers, *GTSE1* exhibited significant clinical significance in LUAD. Kaplan–Meier curve results suggested an association between a high *GTSE1* expression level and a poor prognosis among LUAD patients (Figure 8). Based on ROC analysis, *GTSE1* expression level showed a potential to effectively differentiate between LUAD and its control tissue (Table 1). Furthermore, baseline data from selected GEO and ArrayExpress datasets (Supporting Information S3) along with the results of multivariate Cox regression analysis (Supporting Information S4) contribute to the investigation of the relationship between GTSE1 and the prognosis of LUAD, elucidating the prognostic significance of GTSE1 in pulmonary adenocarcinoma.

**TABLE 1:** Receiver operating characteristic curve analysis results of *GTSE1* expression to identify normal control and LUAD group.

Dataset	AUC	Cutoff	Sensitivity	Specificity	TP	FP	FN	TN
GSE10072	0.818	6.810	0.788	0.758	26	8	7	25
GSE115002	0.721	4.864	0.538	0.865	28	7	24	45
GSE21933	0.782	4.152	0.952	0.667	20	7	1	14
GSE32665	0.729	8.353	0.793	0.678	69	28	18	59
GSE32867	0.718	7.211	0.672	0.759	39	14	19	44
GSE46539	0.885	0.081	0.815	0.924	75	7	17	85
GSE63459	0.598	7.254	0.469	0.750	15	8	17	24
GSE75037	0.820	5.443	0.663	0.916	55	7	28	76
GSE7670	0.683	8.524	0.481	0.889	13	3	14	24

## 4 DISCUSSION

Up to now, numerous studies have demonstrated that *GTSE1* is a potential risk gene for the development and progression of different tumors, including prostate cancer, BRCA, liver cancer, and others.^13^, ^15^, ^26^ Overexpression of *GTSE1* has been found to inhibit apoptosis in esophageal malignant tumor cells^16^ and promote the growth and metastasis of LUAD cells.^17^ Despite the extensive research on pan‐cancer in the past 5 years, the association between *GTSE1* and various malignant tumors remains unexplored. Hence, this study was aimed to comprehensively analyze gene expression profiles obtained from TCGA and Gene Expression Omnibus (GEO) databases to examine the levels of *GTSE1* expression, its potential prognostic significance, and the extent of immune infiltration in neighboring tissues and cancerous tissues across 33 different types of malignant tumors. The results revealed upregulation of *GTSE1* in 19 tumor tissues, including cervical cancer, suggesting its potential as a common biomarker for multiple tumors. Furthermore, overexpression of *GTSE1* was associated with unfavorable prognosis in various tumor types. Importantly, these conclusions were based on pan‐cancer analysis of large sample data, suggesting g a certain level of reliability.

*GTSE1* may exhibit distinct mechanisms of action in the development of different tumors. Analysis by Wu et al.^27^ demonstrated that *GTSE1* levels in most cancer tissues (27/28, 96.4%) were approximately 100 times higher than in non‐cancerous tissues. Immunohistochemical methods revealed that cells with high expression levels of *GTSE1* exhibited increased expression of N‐cadherin, β‐catenin, and Snail, indicating that *GTSE1* might drive transformation of endothelial cells in hepatocellular carcinoma. Similarly, Liu et al.^28^ analyzed 95 samples of bladder cancer together with 30 normal samples, finding the increased *GTSE1* expression contributed to cell proliferation, migration, and invasion through regulation of the p53/FoxM1/CCNB1 pathway, leading to an unfavorable prognosis in individuals with bladder cancer. To clarify the relationship of elevated expression levels of *GTSE1* with overall survival across different tumor types, we conducted an analysis on the clinical features and overall survival of the included samples. The results demonstrated a positive correlation between AJCC stage and *GTSE1* expression level. Patients with BRCA and seven other forms of carcinoma under the age of 65 exhibited higher *GTSE1* expression levels, whereas young patients with gastric adenocarcinoma had lower levels of *GTSE1*. Interestingly, head and neck squamous cell carcinoma (HNSCC), acute myeloid leukemia (LAML), and lung squamous cell carcinoma (LUSC) showed higher expression levels of *GTSE1* in males compared to females. Additionally, a high GTSE1 expression level was linked to the decreased overall/disease‐specific survival. Lei et al.^29^ developed a predictive model for clear cell renal cell carcinoma (ccRCC) patients, utilizing age, gender, and *GTSE1* expression level, to estimate survival probabilities at 1, 3, and 5 years. They found that increased *GTSE1* expression was associated with lower prognostic survival rates. Therefore, *GTSE1* is generally considered an important prognostic risk factor.

The identification of biomarkers with diagnostic and predictive value is crucial for personalized cancer treatment.^30^ Therefore, we investigated the diagnostic value of *GTSE1* as an independent pan‐cancer biomarker. Using ROC curves, we evaluated the diagnostic sensitivity and specificity of *GTSE1*, which effectively distinguished cancer tissues from normal tissues in 21 types of tumors with high diagnostic efficiency. Immune infiltration exerts a significant impact on tumor development,^31^ although it is not yet fully understood whether *GTSE1* is associated with tumor immune invasion. Some previous studies have explored the link of *GTSE1* and immune‐related cells, particularly in the context of cancer immunotherapy involving immune checkpoint inhibitors (ICIs).^32^ Lei et al.^29^ discovered a correlation between overexpression of *GTSE1* and increased infiltration of immune cells in renal cell carcinoma. Co‐expression analysis revealed a correlation between elevated levels of *GTSE1* and the presence of ICIs, including PDCD1 (PD1), LAG3, and CTLA4. This suggests that GTSE1 expression could potentially serve as an indicator to assess the effectiveness of ICIS. Tan et al.^33^ elucidated the correlation between GTSE1 and stromal score as well as immune score across various cancer types, furnishing pivotal clues for investigating the types and activity levels of immune cells within the tumor microenvironment. Our research results also confirmed a positive connection between the expression level of *GTSE1* and infiltration levels of B cells, CD4 T cells, CD8 T cells, macrophage cells, and dendritic cells in THYM, THCA, and LIHC. Identifying tumor biomarkers that impact immune surveillance is crucial for our understanding of tumor evolution and provides an opportunity to study escape mechanisms.^34^ In this study, we proposed the immunoinfiltration relationship of *GTSE1* in pan‐cancer for the first time, suggesting that enhanced expression of *GTSE1* might significantly contribute to immune escape of tumor cells, thus affecting tumor progression.

For our research, we obtained a total of 485 LUAD tissue samples and their corresponding adjacent normal tissues from the ArrayExpress and GEO databases. The objective was to investigate the levels of *GTSE1* expression in LUAD tissues and assess its potential clinical significance using extensive data obtained from multiple centers. The findings indicated upregulation of *GTSE1* in LUAD tissues, showing good discriminatory potential for this type of cancer. However, the mechanism by which high expression of *GTSE1* leads to a poorer prognosis in LUAD patients is still controversial. Previous studies have clearly demonstrated upregulation of *GTSE1* expression in lung cancer tissues. Nonetheless, Tian et al.^35^ suggested that differentially expressed *GTSE1* was not significantly associated with clinical characteristics and overall survival of patients. Additionally, Cao et al.^36^ made a significant finding that the *GTSE1*/p53/NF‐κB pathway can be regulated by miR‐181a‐5p, leading to inhibition of invasion and migration in lung adenoma. Since the expression of miR‐181a‐5p was low in LUAD cells, *GTSE1* overexpression could potentially enhance the invasive and migratory abilities of LUAD cells. To clarify the relationship between high *GTSE1* expression and the prognosis of LUAD patients, we conducted an analysis on four prognostic datasets from the GEO database, and the results showed that upregulation of *GTSE1* expression was related with a shorter survival in LUAD patients.

To our knowledge, this study comprehensively integrated data from the TCGA, ArrayExpress, and GEO databases, resulting in a notably augmented sample size, thereby bolstering the robustness of our findings. Specifically, we elucidate, for the first time, disparities in the expression profiles of GTSE1 across diverse cancer types concerning clinical parameters, thus providing substantive implications for tailored cancer therapeutics. Moreover, our inquiry not only scrutinizes the interplay among GTSE1 expression, prognosis, and immune infiltration in pan‐cancer contexts but also discloses, for the inaugural time, the heightened diagnostic efficacy of GTSE1 across 21 distinct cancer types. Furthermore, we corroborate the expression patterns of GTSE1 and its clinical relevance in LUAD. Expanding upon this groundwork, our investigation delves into the nuanced association between GTSE1 expression levels and the infiltration dynamics of six immune cell subtypes (B cells, CD4 T cells, CD8 T cells, neutrophils, macrophages, and dendritic cells) in pan‐cancer settings, thereby underscoring the correlation between the infiltration levels of disparate immune cell subtypes and GTSE1 expression, thereby furnishing novel insights into immune evasion mechanisms and anti‐tumor modalities. Nevertheless, there were several limitations in our study. First, in our previous analysis of differential gene expression, no obvious disparities in *GTSE1* expression levels were observed when compared tumor tissues to normal tissues among patients with PAAD and PCPG. Therefore, additional confirmation with a substantial number of cases is necessary to verify the diagnostic and prognostic significance of *GTSE1* in PAAD and PCPG. Second, since our study relied solely on existing data, the bioinformatics findings would need to be confirmed by further experimental investigations. Future research endeavors should focus on unraveling the molecular mechanism of *GTSE1* at a microscopic level. Lastly, although pan‐cancer analysis results supported the relationship of *GTSE1* expression and immune regulation mechanisms, further exploration is of necessity to thoroughly understand its underlying mechanisms.

## 5 CONCLUSIONS

In summary, *GTSE1* showed a potential as a diagnostic and prognostic biomarker for various types of cancers. Its expression was linked to the presence of immune cells within tumors and was correlated with immune cell infiltration. These findings provided a bioinformatics foundation for studying the effect of *GTSE1* on the treatment and microenvironment of tumors. Further biological experiments will be required to validate its functionality and molecular mechanism in future.

## CONFLICT OF INTEREST

The authors declare that they have no known competing financial interest or personal relationships that could have appeared to influence the work reported in this paper.

## AUTHOR CONTRIBUTIONS

Guanqiang Yan, Guosheng Li, and Huafu Zhou conceived and designed the study, data analysis, and article writing. Guanqiang Yan provided contributions to data curation and writing—original draft. Guosheng Li made forms. Xiang Gao finished the data validation and paper revision. Jun Liu and Yue Li downloaded data. Jingxiao Li finished visualization and writing—review & editing. Huafu Zhou was the corresponding author. All persons designated as the authors have participated sufficiently in the work to take public responsibility for the content of the manuscript.

## ETHICS STATEMENT

The study was conducted in accordance with the Declaration of Helsinki, and granted exemption by the First Affiliated Hospital of Guangxi Medical University Ethical Review Committee.

## Supporting information


**Supplementary material S1.** Baseline information of some datasets used to explore the prognosis of GSET1.

**Supplementary material S2.** Multivariate Cox regression analysis results of the prognosis of GSET1.

**Supplementary material S3.** Baseline information of some datasets used to explore the prognosis of GSET1.

**Supplementary material S4.** Multivariate Cox regression analysis results of the prognosis of GSET1.

## Data Availability

All data mentioned in the manuscript can be provided by the corresponding author upon reasonable request.
