# Supplementary material for: GTSE1: A potential prognostic and diagnostic biomarker in various tumors including lung adenocarcinoma
Source: Clin Respir J. 2024 May 7;18(5):e13757. doi: 10.1111/crj.13757 (PMC11077242; doi:10.1111/crj.13757)
Supplement: Supplementary file 1 — Supplementary material S1. Baseline information of some datasets used to explore the prognosis of GSET1. [file CRJ-18-e13757-s001.docx]

| **Supplementary material 1.** Baseline information of some datasets used to explore the prognosis of GSET1. | | | | |
| --- | --- | --- | --- | --- |
| **Cancer type** | **Parameters** | | **Sample number** | |
|  |  |  | **Tumor** | **Normal** |
| ACC | Gender | Female | 46 (59.7%) |  |
|  |  | Male | 31 (40.3%) |  |
|  | Age in year | <65 | 66 (85.7%) |  |
|  |  | ≥65 | 11 (14.3%) |  |
|  | AJCC stage | Stage I | 9 (11.7%) |  |
|  |  | Stage II | 36 (46.8%) |  |
|  |  | Stage III | 15 (19.5%) |  |
|  |  | Stage IV | 15 (19.5%) |  |
|  |  | NA | 2 (2.6%) |  |
| BLCA | Gender | Female | 106 (26%) | 9 (47.4%) |
|  |  | Male | 301 (74%) | 10 (52.6%) |
|  | Age in year | <65 | 150 (36.9%) | 5 (26.3%) |
|  |  | ≥65 | 257 (63.1%) | 14 (73.7%) |
|  | AJCC stage | NA | 2 (0.5%) | 0 (0%) |
|  |  | Stage I | 2 (0.5%) | 0 (0%) |
|  |  | Stage II | 130 (31.9%) | 4 (21.1%) |
|  |  | Stage III | 140 (34.4%) | 7 (36.8%) |
|  |  | Stage IV | 133 (32.7%) | 8 (42.1%) |
| BRCA | Gender | Female | 1079 (98.8%) | 112 (99.1%) |
|  |  | NA | 1 (0.1%) | 0 (0%) |
|  | Age in year | <65 | 748 (68.5%) | 82 (72.6%) |
|  |  | ≥65 | 342 (31.3%) | 31 (27.4%) |
|  |  | NA | 2 (0.2%) | 0 (0%) |
|  |  | Male | 12 (1.1%) | 1 (0.9%) |
|  | AJCC stage | NA | 25 (2.3%) | 1 (0.9%) |
|  |  | Stage I | 182 (16.7%) | 20 (17.7%) |
|  |  | Stage II | 617 (56.5%) | 65 (57.5%) |
|  |  | Stage III | 248 (22.7%) | 25 (22.1%) |
|  |  | Stage IV | 20 (1.8%) | 2 (1.8%) |
| CESC | Age in year | <65 | 265 (87.2%) | 2 (66.7%) |
|  |  | ≥65 | 39 (12.8%) | 1 (33.3%) |
| CHOL | Gender | Female | 20 (55.6%) | 3 (33.3%) |
|  |  | Male | 16 (44.4%) | 6 (66.7%) |
|  | Age in year | <65 | 17 (47.2%) | 1 (11.1%) |
|  |  | ≥65 | 19 (52.8%) | 8 (88.9%) |
|  | AJCC stage | Stage I | 19 (52.8%) | 7 (77.8%) |
|  |  | Stage II | 9 (25%) | 1 (11.1%) |
|  |  | Stage III | 1 (2.8%) | 0 (0%) |
|  |  | Stage IV | 7 (19.4%) | 1 (11.1%) |
| COAD | Gender | Female | 130 (45.1%) | 21 (51.2%) |
|  |  | Male | 156 (54.2%) | 20 (48.8%) |
|  |  | NA | 2 (0.7%) | 0 (0%) |
|  | Age in year | <65 | 125 (43.4%) | 11 (26.8%) |
|  |  | ≥65 | 161 (55.9%) | 30 (73.2%) |
|  |  | NA | 2 (0.7%) | 0 (0%) |
|  | AJCC stage | Stage I | 44 (15.3%) | 4 (9.8%) |
|  |  | Stage II | 110 (38.2%) | 22 (53.7%) |
|  |  | Stage III | 82 (28.5%) | 7 (17.1%) |
|  |  | Stage IV | 40 (13.9%) | 7 (17.1%) |
|  |  | NA | 12 (4.2%) | 1 (2.4%) |
| DLBC | Gender | Female | 25 (53.2%) |  |
|  |  | Male | 22 (46.8%) |  |
|  | Age in year | <65 | 33 (70.2%) |  |
|  |  | ≥65 | 14 (29.8%) |  |
| ESCA | Gender | Female | 26 (14.4%) | 5 (38.5%) |
|  |  | Male | 155 (85.6%) | 8 (61.5%) |
|  | Age in year | <65 | 104 (57.5%) | 5 (38.5%) |
|  |  | ≥65 | 77 (42.5%) | 8 (61.5%) |
|  | AJCC stage | Stage I | 18 (9.9%) | 6 (46.2%) |
|  |  | Stage II | 76 (42%) | 3 (23.1%) |
|  |  | Stage III | 55 (30.4%) | 2 (15.4%) |
|  |  | Stage IV | 9 (5%) | 0 (0%) |
|  |  | NA | 23 (12.7%) | 2 (15.4%) |
| GBM | Gender | Female | 54 (35.3%) | 0 (0%) |
|  |  | Male | 98 (64.1%) | 0 (0%) |
|  |  | NA | 1 (0.7%) | 5 (100%) |
|  | Age in year | <65 | 96 (62.7%) | 0 (0%) |
|  |  | ≥65 | 56 (36.6%) | 0 (0%) |
|  |  | NA | 1 (0.7%) | 5 (100%) |
| HNSCC | Gender | Female | 136 (26.3%) | 14 (31.8%) |
|  |  | Male | 382 (73.7%) | 30 (68.2%) |
|  | Age in year | <65 | 326 (62.9%) | 24 (54.5%) |
|  |  | ≥65 | 191 (36.9%) | 20 (45.5%) |
|  |  | NA | 1 (0.2%) | 0 (0%) |
|  | AJCC stage | Stage I | 27 (5.2%) | 2 (4.5%) |
|  |  | Stage II | 70 (13.5%) | 16 (36.4%) |
|  |  | Stage III | 80 (15.4%) | 8 (18.2%) |
|  |  | Stage IV | 266 (51.4%) | 17 (38.6%) |
|  |  | NA | 75 (14.5%) | 1 (2.3%) |
| KICH | Gender | Female | 27 (40.9%) | 12 (48%) |
|  |  | Male | 39 (59.1%) | 13 (52%) |
|  | Age in year | <65 | 51 (77.3%) | 18 (72%) |
|  |  | ≥65 | 15 (22.7%) | 7 (28%) |
|  | AJCC stage | Stage I | 21 (31.8%) | 10 (40%) |
|  |  | Stage II | 25 (37.9%) | 8 (32%) |
|  |  | Stage III | 14 (21.2%) | 3 (12%) |
|  |  | Stage IV | 6 (9.1%) | 4 (16%) |
| KIRC | Gender | Female | 186 (35.1%) | 20 (27.8%) |
|  |  | Male | 344 (64.9%) | 52 (72.2%) |
|  | Age in year | <65 | 330 (62.3%) | 41 (56.9%) |
|  |  | ≥65 | 200 (37.7%) | 31 (43.1%) |
|  | AJCC stage | Stage I | 266 (50.2%) | 25 (34.7%) |
|  |  | Stage II | 57 (10.8%) | 11 (15.3%) |
|  |  | Stage III | 123 (23.2%) | 16 (22.2%) |
|  |  | Stage IV | 81 (15.3%) | 20 (27.8%) |
|  |  | NA | 3 (0.6%) | 0 (0%) |
| KIRP | Gender | Female | 75 (26%) | 10 (31.2%) |
|  |  | Male | 213 (74%) | 22 (68.8%) |
|  | Age in year | <65 | 172 (59.7%) | 16 (50%) |
|  |  | ≥65 | 113 (39.2%) | 16 (50%) |
|  |  | NA | 3 (1%) | 0 (0%) |
|  | AJCC stage | Stage I | 170 (59%) | 15 (46.9%) |
|  |  | Stage II | 21 (7.3%) | 1 (3.1%) |
|  |  | Stage III | 52 (18.1%) | 12 (37.5%) |
|  |  | Stage IV | 15 (5.2%) | 4 (12.5%) |
|  |  | NA | 30 (10.4%) | 0 (0%) |
| LAML | Gender | Female | 80 (46.2%) |  |
|  |  | Male | 93 (53.8%) |  |
|  | Age in year | <65 | 120 (69.4%) |  |
|  |  | ≥65 | 53 (30.6%) |  |
| LGG | Gender | Female | 225 (44.2%) |  |
|  |  | Male | 283 (55.6%) |  |
|  |  | NA | 1 (0.2%) |  |
|  | Age in year | <65 | 473 (92.9%) |  |
|  |  | ≥65 | 35 (6.9%) |  |
|  |  | NA | 1 (0.2%) |  |
| LIHC | Gender | Female | 120 (32.5%) | 22 (44%) |
|  |  | Male | 249 (67.5%) | 28 (56%) |
|  | Age in year | <65 | 220 (59.6%) | 20 (40%) |
|  |  | ≥65 | 148 (40.1%) | 30 (60%) |
|  |  | NA | 1 (0.3%) | 0 (0%) |
|  | AJCC stage | Stage I | 169 (45.8%) | 18 (36%) |
|  |  | Stage II | 86 (23.3%) | 11 (22%) |
|  |  | Stage III | 85 (23%) | 12 (24%) |
|  |  | Stage IV | 5 (1.4%) | 1 (2%) |
|  |  | NA | 24 (6.5%) | 8 (16%) |
| LUAD | Gender | Female | 276 (53.8%) | 34 (57.6%) |
|  |  | Male | 237 (46.2%) | 25 (42.4%) |
|  | Age in year | <65 | 220 (42.9%) | 27 (45.8%) |
|  |  | ≥65 | 274 (53.4%) | 32 (54.2%) |
|  |  | NA | 19 (3.7%) | 0 (0%) |
|  | AJCC stage | Stage I | 274 (53.4%) | 30 (50.8%) |
|  |  | Stage II | 122 (23.8%) | 13 (22%) |
|  |  | Stage III | 83 (16.2%) | 13 (22%) |
|  |  | Stage IV | 26 (5.1%) | 2 (3.4%) |
|  |  | NA | 8 (1.6%) | 1 (1.7%) |
| LUSC | Gender | Female | 129 (25.9%) | 14 (28%) |
|  |  | Male | 369 (74.1%) | 36 (72%) |
|  | Age in year | <65 | 169 (33.9%) | 16 (32%) |
|  |  | ≥65 | 320 (64.3%) | 34 (68%) |
|  |  | NA | 9 (1.8%) | 0 (0%) |
|  | AJCC stage | Stage I | 242 (48.6%) | 27 (54%) |
|  |  | Stage II | 161 (32.3%) | 17 (34%) |
|  |  | Stage III | 84 (16.9%) | 5 (10%) |
|  |  | Stage IV | 7 (1.4%) | 1 (2%) |
|  |  | NA | 4 (0.8%) | 0 (0%) |
| MESO | Gender | Female | 16 (18.4%) |  |
|  |  | Male | 71 (81.6%) |  |
|  | Age in year | <65 | 45 (51.7%) |  |
|  |  | ≥65 | 42 (48.3%) |  |
|  | AJCC stage | Stage I | 10 (11.5%) |  |
|  |  | Stage II | 16 (18.4%) |  |
|  |  | Stage III | 45 (51.7%) |  |
|  |  | Stage IV | 16 (18.4%) |  |
| OV | Age in year | <65 | 274 (65.4%) |  |
|  |  | ≥65 | 145 (34.6%) |  |
| PAAD | Gender | Female | 80 (44.9%) | 2 (50%) |
|  |  | Male | 98 (55.1%) | 2 (50%) |
|  | Age in year | <65 | 82 (46.1%) | 3 (75%) |
|  |  | ≥65 | 96 (53.9%) | 1 (25%) |
|  | AJCC stage | Stage I | 21 (11.8%) | 0 (0%) |
|  |  | Stage II | 147 (82.6%) | 4 (100%) |
|  |  | Stage III | 3 (1.7%) | 0 (0%) |
|  |  | Stage IV | 4 (2.2%) | 0 (0%) |
|  |  | NA | 3 (1.7%) | 0 (0%) |
| PCPG | Gender | Female | 100 (56.5%) | 1 (33.3%) |
|  |  | Male | 77 (43.5%) | 2 (66.7%) |
|  | Age in year | <65 | 153 (86.4%) | 3 (100%) |
|  |  | ≥65 | 24 (13.6%) | 0 (0%) |
| PRAD | Age in year | <65 | 329 (66.5%) | 34 (65.4%) |
|  |  | ≥65 | 166 (33.5%) | 18 (34.6%) |
| READ | Gender | Female | 42 (45.7%) | 7 (70%) |
|  |  | Male | 49 (53.3%) | 3 (30%) |
|  |  | NA | 1 (1.1%) | 0 (0%) |
|  | Age in year | <65 | 47 (51.1%) | 5 (50%) |
|  |  | ≥65 | 44 (47.8%) | 5 (50%) |
|  |  | NA | 1 (1.1%) | 0 (0%) |
|  | AJCC stage | Stage I | 12 (13%) | 4 (40%) |
|  |  | Stage II | 24 (26.1%) | 2 (20%) |
|  |  | Stage III | 33 (35.9%) | 2 (20%) |
|  |  | Stage IV | 13 (14.1%) | 2 (20%) |
|  |  | NA | 10 (10.9%) | 0 (0%) |
| SARC | Gender | Female | 141 (54.7%) | 1 (50%) |
|  |  | Male | 117 (45.3%) | 1 (50%) |
|  | Age in year | <65 | 151 (58.5%) | 2 (100%) |
|  |  | ≥65 | 107 (41.5%) | 0 (0%) |
| SKCM | Gender | Female | 42 (41.2%) | 0 (0%) |
|  |  | Male | 60 (58.8%) | 1 (100%) |
|  | Age in year | <65 | 49 (48%) | 1 (100%) |
|  |  | ≥65 | 53 (52%) | 0 (0%) |
|  | AJCC stage | Stage I | 2 (2%) | 0 (0%) |
|  |  | Stage II | 66 (64.7%) | 0 (0%) |
|  |  | Stage III | 26 (25.5%) | 0 (0%) |
|  |  | Stage IV | 3 (2.9%) | 0 (0%) |
|  |  | NA | 5 (4.9%) | 1 (100%) |
| STAD | Gender | Female | 146 (35.3%) | 13 (36.1%) |
|  |  | Male | 268 (64.7%) | 23 (63.9%) |
|  | Age in year | <65 | 173 (41.8%) | 13 (36.1%) |
|  |  | ≥65 | 236 (57%) | 23 (63.9%) |
|  |  | NA | 5 (1.2%) | 0 (0%) |
|  | AJCC stage | Stage I | 58 (14%) | 6 (16.7%) |
|  |  | Stage II | 121 (29.2%) | 19 (52.8%) |
|  |  | Stage III | 169 (40.8%) | 7 (19.4%) |
|  |  | Stage IV | 41 (9.9%) | 3 (8.3%) |
|  |  | NA | 25 (6%) | 1 (2.8%) |
| TGCT | Gender | NA | 16 (10.8%) |  |
|  |  | Male | 132 (89.2%) |  |
|  | Age in year | <65 | 130 (87.8%) |  |
|  |  | ≥65 | 2 (1.4%) |  |
|  |  | NA | 16 (10.8%) |  |
|  | AJCC stage | Stage I | 53 (35.8%) |  |
|  |  | Stage II | 12 (8.1%) |  |
|  |  | Stage III | 14 (9.5%) |  |
|  |  | NA | 69 (46.6%) |  |
| THCA | Gender | Female | 368 (73%) | 42 (71.2%) |
|  |  | Male | 136 (27%) | 17 (28.8%) |
|  | Age in year | <65 | 428 (84.9%) | 49 (83.1%) |
|  |  | ≥65 | 76 (15.1%) | 10 (16.9%) |
|  | AJCC stage | Stage I | 283 (56.2%) | 36 (61%) |
|  |  | Stage II | 52 (10.3%) | 7 (11.9%) |
|  |  | Stage III | 112 (22.2%) | 12 (20.3%) |
|  |  | Stage IV | 55 (10.9%) | 4 (6.8%) |
|  |  | NA | 2 (0.4%) | 0 (0%) |
| THYM | Gender | Female | 57 (47.9%) | 2 (100%) |
|  |  | Male | 62 (52.1%) | 0 (0%) |
|  | Age in year | <65 | 76 (63.9%) | 2 (100%) |
|  |  | ≥65 | 42 (35.3%) | 0 (0%) |
|  |  | NA | 1 (0.8%) | 0 (0%) |
| UCEC | Gender | Female | 180 (100%) | 13 (56.5%) |
|  |  | NA | 0 (0%) | 10 (43.5%) |
|  | Age in year | <65 | 84 (46.7%) | 10 (43.5%) |
|  |  | ≥65 | 93 (51.7%) | 3 (13%) |
|  |  | NA | 3 (1.7%) | 10 (43.5%) |
| UCS | Age in year | <65 | 17 (29.8%) |  |
|  |  | ≥65 | 40 (70.2%) |  |
| UVM | Gender | Female | 35 (44.3%) |  |
|  |  | Male | 44 (55.7%) |  |
|  | Age in year | <65 | 45 (57%) |  |
|  |  | ≥65 | 34 (43%) |  |
|  | AJCC stage | Stage II | 39 (49.4%) |  |
|  |  | Stage III | 35 (44.3%) |  |
|  |  | Stage IV | 4 (5.1%) |  |
|  |  | NA | 1 (1.3%) |  |
