# Supplementary material for: GTSE1: A potential prognostic and diagnostic biomarker in various tumors including lung adenocarcinoma
Source: Clin Respir J. 2024 May 7;18(5):e13757. doi: 10.1111/crj.13757 (PMC11077242; doi:10.1111/crj.13757)
Supplement: Supplementary file 2 — Supplementary material S2. Multivariate Cox regression analysis results of the prognosis of GSET1. [file CRJ-18-e13757-s004.docx]

| **Supplementary material 2.** Multivariate Cox regression analysis results of the prognosis of GSET1. | | | | |
| --- | --- | --- | --- | --- |
| **Dataset** | **Parameters** | | **Hazard ratio** | **P value** |
| ACC | GTSE1 |  | 3.06 (1.83-5.13) | <0.001 |
|  | Age in year | <65 |  |  |
|  |  | 65_ | 2.23 (0.78-6.33) | 0.133 |
|  | Gender | Female |  |  |
|  |  | Male | 1.37 (0.59-3.17) | 0.46 |
|  | AJCC stage | Stage I |  |  |
|  |  | Stage II | 2.25 (0.27-18.93) | 0.456 |
|  |  | Stage III | 5.59 (0.63-49.47) | 0.122 |
|  |  | Stage IV | 5.54 (0.57-53.53) | 0.139 |
| BLCA | GTSE1 |  | 1.01 (0.85-1.20) | 0.929 |
|  | Age in year | <65 |  |  |
|  |  | 65_ | 1.98 (1.40-2.78) | <0.001 |
|  | Gender | Female |  |  |
|  |  | Male | 0.83 (0.60-1.15) | 0.267 |
|  | AJCC stage | Stage I |  |  |
|  |  | Stage II | 2201830.73 (0.00-Inf) | 0.994 |
|  |  | Stage III | 3334995.59 (0.00-Inf) | 0.994 |
|  |  | Stage IV | 6302515.67 (0.00-Inf) | 0.994 |
| BRCA | GTSE1 |  | 1.19 (1.00-1.41) | 0.051 |
|  | Age in year | <65 |  |  |
|  |  | 65_ | 2.66 (1.88-3.77) | <0.001 |
|  | Gender | Female |  |  |
|  |  | Male | 0.57 (0.08-4.07) | 0.572 |
|  | AJCC stage | Stage I |  |  |
|  |  | Stage II | 1.68 (0.97-2.91) | 0.065 |
|  |  | Stage III | 3.39 (1.91-6.04) | <0.001 |
|  |  | Stage IV | 14.93 (7.34-30.36) | <0.001 |
| CESC | GTSE1 |  | 0.98 (0.73-1.32) | 0.895 |
|  | Age in year | <65 |  |  |
|  |  | 65_ | 2.15 (1.23-3.75) | 0.007 |
| CHOL | GTSE1 |  | 1.35 (0.78-2.34) | 0.281 |
|  | Age in year | <65 |  |  |
|  |  | 65_ | 1.41 (0.50-3.99) | 0.513 |
|  | Gender | Female |  |  |
|  |  | Male | 1.23 (0.42-3.55) | 0.707 |
|  | AJCC stage | Stage I |  |  |
|  |  | Stage II | 2.47 (0.73-8.36) | 0.146 |
|  |  | Stage III | 0.00 (0.00-Inf) | 0.998 |
|  |  | Stage IV | 2.48 (0.76-8.03) | 0.131 |
| COAD | GTSE1 |  | 0.95 (0.65-1.39) | 0.788 |
|  | Age in year | <65 |  |  |
|  |  | 65_ | 1.97 (1.13-3.42) | 0.016 |
|  | Gender | Female |  |  |
|  |  | Male | 1.20 (0.72-2.01) | 0.49 |
|  | AJCC stage | Stage I |  |  |
|  |  | Stage II | 2.02 (0.60-6.84) | 0.258 |
|  |  | Stage III | 3.92 (1.17-13.14) | 0.027 |
|  |  | Stage IV | 10.84 (3.17-37.06) | <.001 |
| DLBC | GTSE1 |  | 0.73 (0.36-1.51) | 0.4 |
|  | Age in year | <65 |  |  |
|  |  | 65_ | 0.77 (0.15-3.95) | 0.754 |
|  | Gender | Female |  |  |
|  |  | Male | 1.00 (0.24-4.17) | 0.999 |
| ESCA | GTSE1 |  | 0.93 (0.68-1.27) | 0.641 |
|  | Age in year | <65 |  |  |
|  |  | 65_ | 1.32 (0.79-2.22) | 0.294 |
|  | Gender | Female |  |  |
|  |  | Male | 1.74 (0.68-4.44) | 0.249 |
|  | AJCC stage | Stage I |  |  |
|  |  | Stage II | 2.07 (0.71-6.08) | 0.184 |
|  |  | Stage III | 4.55 (1.50-13.85) | 0.008 |
|  |  | Stage IV | 11.06 (3.11-39.40) | <.001 |
| GBM | GTSE1 |  | 1.02 (0.83-1.24) | 0.858 |
|  | Age in year | <65 |  |  |
|  |  | 65_ | 1.58 (1.09-2.31) | 0.017 |
|  | Gender | Female |  |  |
|  |  | Male | 0.93 (0.63-1.36) | 0.694 |
| HNSCC | GTSE1 |  | 1.06 (0.88-1.28) | 0.537 |
|  | Age in year | <65 |  |  |
|  |  | 65_ | 1.47 (1.09-1.98) | 0.012 |
|  | Gender | Female |  |  |
|  |  | Male | 0.76 (0.55-1.04) | 0.091 |
|  | AJCC stage | Stage I |  |  |
|  |  | Stage II | 1.99 (0.69-5.74) | 0.2 |
|  |  | Stage III | 2.39 (0.84-6.84) | 0.104 |
|  |  | Stage IV | 3.84 (1.42-10.41) | 0.008 |
| KICH | GTSE1 |  | 2.70 (1.55-4.69) | <.001 |
|  | Age in year | <65 |  |  |
|  |  | 65_ | 2.26 (0.57-8.96) | 0.247 |
|  | Gender | Female |  |  |
|  |  | Male | 0.58 (0.14-2.44) | 0.455 |
|  | AJCC stage | Stage I |  |  |
|  |  | Stage II | 10438868.37 (1943122.82-56079817.30) | <.001 |
|  |  | Stage III | 76646780.30 (16245220.45-361628144.59) | <.001 |
|  |  | Stage IV | 493477200.80 (85124116.19-2860760952.47) | <.001 |
| KIRC | GTSE1 |  | 1.73 (1.37-2.19) | <.001 |
|  | Age in year | <65 |  |  |
|  |  | 65_ | 1.73 (1.28-2.35) | <.001 |
|  | Gender | Female |  |  |
|  |  | Male | 0.96 (0.69-1.31) | 0.779 |
|  | AJCC stage | Stage I |  |  |
|  |  | Stage II | 1.21 (0.65-2.25) | 0.549 |
|  |  | Stage III | 2.23 (1.48-3.36) | <.001 |
|  |  | Stage IV | 5.34 (3.59-7.94) | <.001 |
| KIRP | GTSE1 |  | 3.09 (1.97-4.86) | <.001 |
|  | Age in year | <65 |  |  |
|  |  | 65_ | 1.25 (0.64-2.45) | 0.507 |
|  | Gender | Female |  |  |
|  |  | Male | 0.73 (0.36-1.49) | 0.388 |
|  | AJCC stage | Stage I |  |  |
|  |  | Stage II | 0.72 (0.16-3.28) | 0.667 |
|  |  | Stage III | 2.36 (1.05-5.33) | 0.038 |
|  |  | Stage IV | 9.90 (4.01-24.41) | <.001 |
| LAML | GTSE1 |  | 1.06 (0.83-1.35) | 0.644 |
|  | Age in year | <65 |  |  |
|  |  | 65_ | 3.35 (2.25-4.99) | <.001 |
|  | Gender | Female |  |  |
|  |  | Male | 0.87 (0.58-1.30) | 0.496 |
| LGG | GTSE1 |  | 1.60 (1.37-1.86) | <.001 |
|  | Age in year | <65 |  |  |
|  |  | 65_ | 4.27 (2.51-7.27) | <.001 |
|  | Gender | Female |  |  |
|  |  | Male | 1.06 (0.74-1.53) | 0.742 |
| LIHC | GTSE1 |  | 1.50 (1.24-1.82) | <.001 |
|  | Age in year | <65 |  |  |
|  |  | 65_ | 1.43 (0.96-2.13) | 0.075 |
|  | Gender | Female |  |  |
|  |  | Male | 1.01 (0.68-1.49) | 0.973 |
|  | AJCC stage | Stage I |  |  |
|  |  | Stage II | 1.34 (0.82-2.22) | 0.246 |
|  |  | Stage III | 2.38 (1.54-3.70) | <.001 |
|  |  | Stage IV | 8.85 (2.57-30.42) | 0.001 |
| LUAD | GTSE1 |  | 1.24 (1.07-1.43) | 0.004 |
|  | Age in year | <65 |  |  |
|  |  | 65_ | 1.33 (0.98-1.81) | 0.068 |
|  | Gender | Female |  |  |
|  |  | Male | 1.03 (0.76-1.39) | 0.842 |
|  | AJCC stage | Stage I |  |  |
|  |  | Stage II | 2.13 (1.47-3.09) | <.001 |
|  |  | Stage III | 3.08 (2.10-4.53) | <.001 |
|  |  | Stage IV | 3.54 (2.02-6.21) | <.001 |
| LUSC | GTSE1 |  | 0.95 (0.80-1.13) | 0.555 |
|  | Age in year | <65 |  |  |
|  |  | 65_ | 1.33 (0.98-1.81) | 0.065 |
|  | Gender | Female |  |  |
|  |  | Male | 1.19 (0.86-1.65) | 0.286 |
|  | AJCC stage | Stage I |  |  |
|  |  | Stage II | 1.21 (0.88-1.68) | 0.243 |
|  |  | Stage III | 1.61 (1.12-2.31) | 0.01 |
|  |  | Stage IV | 2.93 (1.18-7.28) | 0.021 |
| MESO | GTSE1 |  | 2.47 (1.78-3.42) | <.001 |
|  | Age in year | <65 |  |  |
|  |  | 65_ | 1.40 (0.86-2.28) | 0.172 |
|  | Gender | Female |  |  |
|  |  | Male | 0.76 (0.41-1.42) | 0.392 |
|  | AJCC stage | Stage I |  |  |
|  |  | Stage II | 0.81 (0.33-1.95) | 0.633 |
|  |  | Stage III | 0.67 (0.31-1.43) | 0.297 |
|  |  | Stage IV | 0.59 (0.24-1.47) | 0.258 |
| OV | GTSE1 |  |  |  |
|  | Age in year | <65 | 1.04 (0.90-1.19) | 0.607 |
|  |  | 65_ |  |  |
|  |  |  | 1.45 (1.13-1.85) | 0.003 |
| PAAD | GTSE1 |  | 1.31 (0.98-1.75) | 0.067 |
|  | Age in year | <65 |  |  |
|  |  | 65_ | 1.32 (0.86-2.01) | 0.202 |
|  | Gender | Female |  |  |
|  |  | Male | 0.81 (0.53-1.22) | 0.309 |
|  | AJCC stage | Stage I |  |  |
|  |  | Stage II | 1.95 (0.88-4.34) | 0.1 |
|  |  | Stage III | 1.22 (0.15-10.01) | 0.853 |
|  |  | Stage IV | 1.98 (0.40-9.67) | 0.4 |
| PCPG | GTSE1 |  | 1.07 (0.24-4.84) | 0.932 |
|  | Age in year | <65 |  |  |
|  |  | 65_ | 2.12 (0.23-19.36) | 0.507 |
|  | Gender | Female |  |  |
|  |  | Male | 3.24 (0.54-19.58) | 0.2 |
| PRAD | GTSE1 |  | 2.41 (1.01-5.74) | 0.047 |
|  | Age in year | <65 |  |  |
|  |  | 65_ | 2.69 (0.73-9.90) | 0.135 |
| READ | GTSE1 |  | 0.53 (0.14-1.95) | 0.34 |
|  | Age in year | <65 |  |  |
|  |  | 65_ | 10.84 (1.36-86.48) | 0.024 |
|  | Gender | Female |  |  |
|  |  | Male | 0.38 (0.10-1.41) | 0.148 |
|  | AJCC stage | Stage I |  |  |
|  |  | Stage II | 0.21 (0.02-2.42) | 0.209 |
|  |  | Stage III | 0.93 (0.18-4.87) | 0.929 |
|  |  | Stage IV | 1.10 (0.15-8.07) | 0.924 |
| SARC | GTSE1 |  | 1.14 (0.95-1.36) | 0.147 |
|  | Age in year | <65 |  |  |
|  |  | 65_ | 2.11 (1.41-3.14) | <.001 |
|  | Gender | Female |  |  |
|  |  | Male | 0.94 (0.62-1.43) | 0.766 |
| SKCM | GTSE1 |  | 1.12 (0.72-1.74) | 0.624 |
|  | Age in year | <65 |  |  |
|  |  | 65_ | 0.89 (0.37-2.17) | 0.8 |
|  | Gender | Female |  |  |
|  |  | Male | 1.04 (0.46-2.33) | 0.923 |
|  | AJCC stage | Stage I |  |  |
|  |  | Stage II | 41917021.50 (0.00-Inf) | 0.998 |
|  |  | Stage III | 69348248.58 (0.00-Inf) | 0.998 |
|  |  | Stage IV | 381041627.59 (0.00-Inf) | 0.997 |
| STAD | GTSE1 |  | 0.83 (0.69-1.01) | 0.064 |
|  | Age in year | <65 |  |  |
|  |  | 65_ | 1.89 (1.33-2.68) | <.001 |
|  | Gender | Female |  |  |
|  |  | Male | 1.14 (0.80-1.61) | 0.469 |
|  | AJCC stage | Stage I |  |  |
|  |  | Stage II | 1.60 (0.83-3.09) | 0.164 |
|  |  | Stage III | 2.52 (1.37-4.64) | 0.003 |
|  |  | Stage IV | 4.92 (2.44-9.93) | <.001 |
| TGCT | GTSE1 |  | 3.85 (0.07-197.91) | 0.503 |
|  | Age in year | <65 |  |  |
|  |  | 65_ | 54878286.21 (0.00-Inf) | 1 |
|  | AJCC stage | Stage I |  |  |
|  |  | Stage II | 782560580.34 (0.00-Inf) | 0.999 |
|  |  | Stage III | 3747956119754586112.00 (0.00-Inf) | 1 |
| THCA | GTSE1 |  | 1.19 (0.33-4.23) | 0.79 |
|  | Age in year | <65 |  |  |
|  |  | 65_ | 19.18 (4.72-77.99) | <.001 |
|  | Gender | Female |  |  |
|  |  | Male | 1.49 (0.50-4.37) | 0.472 |
|  | AJCC stage | Stage I |  |  |
|  |  | Stage II | 1.92 (0.23-16.03) | 0.549 |
|  |  | Stage III | 1.96 (0.33-11.54) | 0.457 |
|  |  | Stage IV | 5.87 (1.00-34.50) | 0.05 |
| THYM | GTSE1 |  | 0.60 (0.35-1.03) | 0.065 |
|  | Age in year | <65 |  |  |
|  |  | 65_ | 1.76 (0.36-8.53) | 0.483 |
|  | Gender | Female |  |  |
|  |  | Male | 0.48 (0.11-2.12) | 0.336 |
| UCEC | GTSE1 |  | 0.93 (0.66-1.31) | 0.679 |
|  | Age in year | <65 |  |  |
|  |  | 65_ | 0.93 (0.46-1.87) | 0.844 |
| UCS | GTSE1 |  | 0.77 (0.44-1.35) | 0.363 |
|  | Age in year | <65 |  |  |
|  |  | 65_ | 1.12 (0.51-2.46) | 0.782 |
| UVM | GTSE1 |  | 4.39 (1.67-11.53) | 0.003 |
|  | Age in year | <65 |  |  |
|  |  | 65_ | 2.23 (0.90-5.51) | 0.082 |
|  | Gender | Female |  |  |
|  |  | Male | 2.19 (0.82-5.85) | 0.117 |
|  | AJCC stage | Stage II |  |  |
|  |  | Stage III | 0.95 (0.36-2.48) | 0.917 |
|  |  | Stage IV | 57.75 (5.40-617.05) | 0.001 |
