# Supplementary material for: GTSE1: A potential prognostic and diagnostic biomarker in various tumors including lung adenocarcinoma
Source: Clin Respir J. 2024 May 7;18(5):e13757. doi: 10.1111/crj.13757 (PMC11077242; doi:10.1111/crj.13757)
Supplement: Supplementary file 3 — Supplementary material S3. Baseline information of some datasets used to explore the prognosis of GSET1. [file CRJ-18-e13757-s002.docx]

| **Supplementary material 3.** Baseline information of some datasets used to explore the prognosis of GSET1. | | | |
| --- | --- | --- | --- |
| **Dataset** | **Parameters** | | **Sample number (%)** |
| GSE11969 | Gender | female | 48 (29.4%) |
|  |  | male | 101 (62.0%) |
|  |  | Sex: NA | 14 (8.6%) |
|  | Age in year | <65 | 87 (58.4%) |
|  |  | ≥65 | 62 (41.6%) |
|  | TNM stage | I | 78 (47.9%) |
|  |  | II | 26 (16.0%) |
|  |  | III | 45 (27.6%) |
|  |  | N | 14 (8.6%) |
| GSE13213 | Gender | F | 57 (48.7%) |
|  |  | M | 60 (51.3%) |
|  | Age in year | <65 | 76 (65.0%) |
|  |  | ≥65 | 41 (35.0%) |
|  | TNM stage | I | 79 (67.5%) |
|  |  | II | 13 (11.1%) |
|  |  | III | 25 (21.4%) |
| GSE14814 | Gender | Female | 42 (31.6%) |
|  |  | Male | 91 (68.4%) |
|  | Age in year | <65 | 87 (65.4%) |
|  |  | ≥65 | 46 (34.6%) |
|  | TNM stage | I | 73 (54.9%) |
|  |  | II | 60 (45.1%) |
