# Supplementary material for: GTSE1: A potential prognostic and diagnostic biomarker in various tumors including lung adenocarcinoma
Source: Clin Respir J. 2024 May 7;18(5):e13757. doi: 10.1111/crj.13757 (PMC11077242; doi:10.1111/crj.13757)
Supplement: Supplementary file 4 — Supplementary material S4. Multivariate Cox regression analysis results of the prognosis of GSET1. [file CRJ-18-e13757-s003.docx]

| **Supplementary material 4.** Multivariate Cox regression analysis results of the prognosis of GSET1. | | | | |
| --- | --- | --- | --- | --- |
| **Dataset** | **Parameters** | | **Hazard ratio** | **P value** |
| GSE11969 | GTSE1 | | 1.65 (0.19-14.60) | 0.654 |
|  | Age in year | <65 |  |  |
|  |  | ≥65 | 1.63 (1.01-2.63) | 0.044 |
|  | Gender | female |  |  |
|  |  | male | 1.42 (0.84-2.39) | 0.189 |
|  | TNM stage | I |  |  |
|  |  | II | 1.70 (0.84-3.46) | 0.142 |
|  |  | III | 4.02 (2.39-6.76) | <.0001 |
| GSE13213 | GTSE1 | | 1.86 (1.17-2.98) | 0.009 |
|  | Age in year | <65 |  |  |
|  |  | ≥65 | 1.35 (0.75-2.44) | 0.313 |
|  | Gender | F |  |  |
|  |  | M | 1.32 (0.74-2.36) | 0.353 |
|  | TNM stage | I |  |  |
|  |  | II | 1.82 (0.72-4.58) | 0.202 |
|  |  | III | 3.40 (1.84-6.30) | <0.001 |
| GSE14814 | GTSE1 | | 1.08 (0.38-3.08) | 0.891 |
|  | Age in year | <65 |  |  |
|  |  | ≥65 | 1.53 (0.70-3.35) | 0.291 |
|  | Gender | Female |  |  |
|  |  | Male | 1.90 (0.94-3.88) | 0.076 |
|  | TNM stage | I |  |  |
|  |  | II | 1.91 (0.95-3.84) | 0.071 |
